# A Comparative Analysis of Friction and Energy Losses in Hydrogen and CNG Fueled Engines: Implications on the Top Compression Ring Design Using Steel, Cast Iron, and Silicon Nitride Materials

**DOI:** 10.3390/ma17153806

**Published:** 2024-08-01

**Authors:** Vasiliki-Ioanna Nikolopoulou, Anastasios Zavos, Pantelis Nikolakopoulos

**Affiliations:** Machine Design Laboratory, Department of Mechanical Engineering and Aeronautics, University of Patras, 26504 Patras, Greece; up1054527@ac.upatras.gr (V.-I.N.); zavos@upatras.gr (A.Z.)

**Keywords:** top compression ring, compressed natural gas (CNG), hydrogen fuel, blow-by, fuel delivery system, energy loss

## Abstract

Optimizing the design of the top compression ring holds immense importance in reducing friction across both traditional Internal Combustion (IC) engines and hybrid power systems. This study investigates the impact of alternative fuels, specifically hydrogen and CNG, on the behavior of top piston rings within internal combustion (IC) engines. The goal of this approach is to understand the complex interplay between blow-by, fuel type, material behavior, and their effects on ring friction, energy losses, and resulting ring strength. Two types of IC engines were analyzed, taking into account flow conditions derived from in-cylinder pressures and piston geometry. Following ISO 6622-2:2013 guidelines, thick top compression rings made from varying materials (steel, cast iron, and silicon nitride) were investigated and compared. Through a quasi-static ring model within Computational Fluid Dynamics (CFD), critical tribological parameters such as the minimum film and ring friction were simulated, revealing that lighter hydrogen-powered engines with higher combustion pressures could potentially experience approximately 34.7% greater power losses compared to their heavier CNG counterparts. By delving into the interaction among the fuel delivery system, gas blow-by, and material properties, this study unveils valuable insights into the tribological and structural behavior of the top piston ring conjunction. Notably, the silicon nitride material demonstrates promising strength improvements, while the adoption of Direct Injection (DI) is associated with approximately 10.1% higher energy losses compared to PFI. Such findings carry significant implications for enhancing engine efficiency and promoting sustainable energy utilization.

## 1. Introduction

The automotive industry is currently undergoing a profound transformation aimed at enhancing internal combustion (IC) engines to align with stringent environmental standards, such as EURO 6, which regulates the levels of harmful emissions in exhaust gases. This convergence in standards sets the stage for a more unified approach to future regulations, potentially exemplified by the forthcoming EURO 7 standard [1]. Therefore, ongoing scientific research endeavors are focused on the refinement and modernization of internal combustion engines [2].

Zero-emission vehicles represent a top priority for engine manufacturers, and gaseous fuels like compressed natural gas (CNG) and hydrogen have shown promising potential in reducing CO_2_ emissions in the transportation sector [3,4,5]. Notably, the utilization of CNG as a fuel source has led to reduced atmospheric pollutants in India, a region characterized by high transportation sector activity [6]. Additionally, hydrogen-fueled engines have found diverse applications, ranging from buses and trucks to experimental passenger cars. These engines exhibit particular suitability for heavy-duty vehicles, offering extended driving ranges and shorter refueling times. Moreover, the combustion of hydrogen within conventional internal combustion (IC) systems presents its own set of difficulties. These include adverse effects on metallic components, such as embrittlement and excessive wear [7]. Hydrogen combustion can also hasten the deterioration of engine lubricants, leading to a reduced operational lifespan when compared to their hydrocarbon-fueled counterparts [8]. This research delves into the impacts of both CNG and hydrogen fuels on the performance of the top piston ring within two distinct engine systems. 

It is widely recognized that the top compression ring plays a critical role in engine efficiency and losses [9]. Specifically, the ring-pack assembly is the primary source of friction, and according to Richardson [10], the top compression ring is the main source of frictional losses, causing between 13% and 40% of these losses, depending on the engine type and running conditions. Therefore, reducing ring frictional losses by 10–50% under boundary-lubricated conditions [11] would enhance fuel efficiency, which is crucial for engine downsizing. Several studies have endeavored to precisely predict these losses, employing diverse levels of analytical complexity. Early investigations, pioneered by Castleman [12] and Furuhama [13], presented film thickness in ring–liner contact, particularly around reversal points. Furthermore, Akalin and Newaz [14] provided numerical results that concurred with the experimental findings of Furuhama and Sasaki [15]. The evolution of analyses then encompassed FEM and CFD examinations. Baelden and Tian [16] introduced finite element analysis (FEA) to explore the effect of in-cylinder ring conformability and structural deformation during oil flow. Shahmohamadi et al. [17] introduced a CFD methodology for solving the Navier–Stokes equations and compared their solutions with those derived from the Reynolds equation and the Elrod cavitation model. Zavos and Nikolakopoulos [18] predicted lubricant film thickness and generated friction under different engine speeds and compression ring curvature heights, utilizing a CFD code that, under quasi-static conditions, solves the Navier–Stokes equations while assuming an isothermal state. Incorporating temperature effects, Morris et al. [19] compared results under isothermal and variable oil temperature conditions. Their findings demonstrated that variable oil temperature led to shear thinning of the lubricant film thickness and, consequently, reduced energy losses compared to isothermal conditions. Lastly, Gore M. et al. [20] and Zavos [21] explored the topography, geometry, and materials of compression rings for various loads and speeds. Their investigation encompassed plated and unplated steel compression rings, with measurements including friction coefficients, micro-roughness loads, and hydrodynamic pressure under boundary and mixed lubrication regimes. 

In terms of functional requirements, the top compression ring necessitates superior mechanical, thermal and, above all, tribological resistance. This demand has led to the utilization of various materials characterized by a high modulus of elasticity and bending strength. For enhanced fatigue strength, durability, and superior wear resistance, options include high chromium alloyed steels [22], heat-treated low-alloyed nodular cast irons [23], and composite materials utilizing an aluminum alloy matrix [24]. To further bolster their mechanical strength and wear resistance, diverse coatings have been applied to these structural materials. Zabala et al. [25] examined several coated piston rings, encompassing different CrN PVD coatings as well as self-lubricious diamond-like carbon and MoS2 coatings, aiming for optimal performance in terms of wear and fuel consumption. Wróblewski [26] and Dziubak et al. [27] studied and reviewed the effects of anti-wear coatings in ring–liner conjunction, exploring how factors such as inlet air pollution and the microstructure of coatings influence wear performance. Dolatabadi et al. [28] delved into the impacts of coatings on ring and cylinder surfaces, emphasizing their significance in enhancing engine fuel economy through multi-physics modeling of ring dynamics, contact tribology, and thermal effects. Addressing these intricate phenomena necessitates the use of finite element methods to comprehend ring strength and thermo-chemical changes. Mishra et al. [29] developed an FEM model incorporating TiSiCN-coated rings within a piston assembly, exploring diverse piston crown geometries to discern variations in ring strength properties. Similarly, Chen et al. [30] constructed a 3D model illustrating the influence of ring structure on sealing behavior. Thus, an interactive model elucidating the tribodynamic analysis of piston ring–liner contacts and strength performance is indispensable in powertrains to surmount and gain deeper insights into energy efficiency and durability challenges.

Recently, the influence of alternative fuels on lubricated engine components has garnered significant attention in research. Antunes et al. [31] proposed that, given the elevated combustion pressures in hydrogen-fueled engines, it was prudent to prioritize the development of more robust engine components, including the piston pin, piston, and piston rings. Balyts’kyi et al. [32] introduced a model addressing the phenomenon of blowout within a piston assembly system across various engine types. Their findings underscore the substantial impact of hydrogen fuel on the sealing characteristics of piston rings. A detailed exploration into the impact of hydrogen on piston ring wear was also undertaken by Kindrachuk et al. [33]. Their investigation showed that hydrogen generation could occur as a consequence of the breakdown or decomposition of lubricants exposed to the elevated temperatures and pressures within the combustion chamber. Subsequently, this could lead to hydrogen diffusion into the solid material structure, primarily driven by the energy imparted during sliding friction. Additionally, the tribological effects of utilizing Port Injection (PI) and Direct Injection (DI) hydrogen-powered internal combustion (IC) engines were recently presented by Rahmani et al. [34]. Their study revealed a potential reduction in boundary friction and cylinder temperature with the use of a PFI hydrogen engine. A relatively unexplored aspect in the topic of Hydrogen-fueled Internal Combustion Engines (HICEs) revolves around the impact of hydrogen combustion on the tribological behavior of in-cylinder components. It becomes evident that the operational conditions within the engine markedly influence the contact pressures and temperatures generated during combustion [35].

The method presented here serves as a tool for predicting compression ring power and energy losses in two distinct engine systems—CNG and hydrogen—as fuels. By utilizing empirically measured combustion pressures and analyzing blow-by phenomena within analytical quasi-static modeling methodologies, this model provides a platform for realistically assessing flow dynamics near the top piston ring. In accordance with ISO 6622-2:2013 [36] guidelines, thick top compression rings were modeled in both engines using steel, cast iron, and silicon nitride as base materials. To ensure comparability, all simulations in this work utilize the same SAE 10W40 lubricant. This approach enables the discernment of the complex interplay between blow-by, fuel characteristics, and material properties. Additionally, this integrated study of ring tribology and structural analysis represents original contributions, specifically exploring the effects of different fuel delivery systems in hydrogen engines.

## 2. Materials and Methods

### 2.1. Gas Blow-by Model

**Figure 1:**
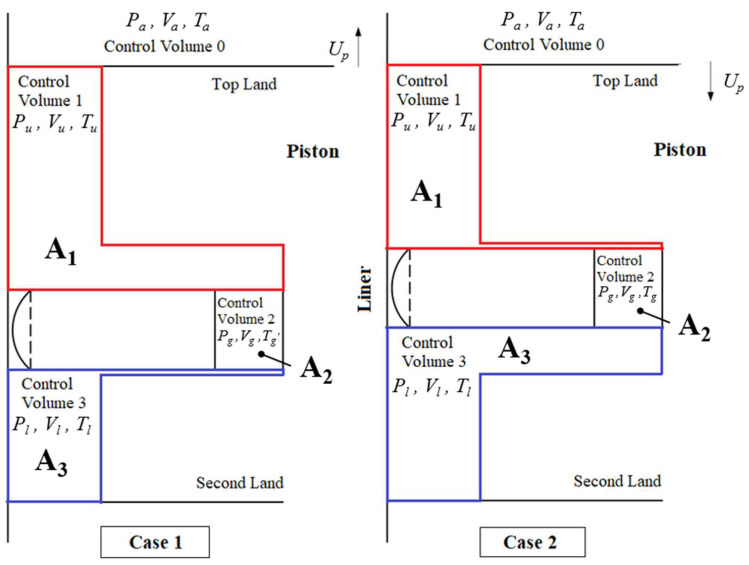
Simplified control volume model for the gas blow-by phenomenon through the top compression ring.

Figure 1 illustrates a simplified control volume model for gas blow-by through the top compression ring, based on the method developed by Baker et al. [37], which incorporates techniques from Gulwadi [38] and Ruddy et al. [39]. 

In Figure 1, the summary of the gas blow-by modeling methodology includes an explanation of the control volumes and their characteristics, such as pressure, volume, and temperature, with the following parameters:

- *A*_1_, *A*_2_ and *A*_3_ are the cross-sectional areas of control volumes 1, 2 and 3;
- *P_u_*, *P_g_*, and *P_l_*, are the gas pressures in control volumes 1, 2 and 3;
- *V_u_*, *V_g_*, and *V_l_*, are the volumes in control volumes 1, 2 and 3;
- *T_u_*, *T_g_*, and *T_l_*, are the gas temperatures in control volumes 1, 2 and 3;
- *P_a_*, *V_a_*, and *T_a_*, are the in-cylinder pressure, volume and temperature.

The gas blow-by modeling methodology is summarized as follows:

- **Case 1**—The first compression ring is assumed to be held firmly against the bottom groove lands within the piston groove, without fluttering, due to axial forces and pressure differentials. This position is maintained as the piston moves upward from BDC to TDC during the compression stroke (−180° ≤ φ < 0°).
- **Case 2**—The first compression ring is also assumed to remain almost pressed against the top groove lands inside the piston groove as the piston moves downward from TDC to BDC during the power stroke (0° ≤ φ < 180°). Dynamic phenomena such as piston ring flutter and collapse are disregarded;
- Combustion products are treated as ideal gases and follow the ideal gas law [40]. Namazian and Heywood [41] experimentally confirmed that the Reynolds number for gas mass flow in control volumes 1, 2, and 3 is relatively low (Re ≈ 10), validating the assumption of laminar flow for the gas blow-by problem. The combustion chamber to control volume 1 flow is considered fully laminar, resembling a Couette flow situation. Analyses of the crevice pressure drop have revealed it to be minimal, typically within 0.1–0.2% for standard production engine crevice sizes. Thus, we can assume that control volume 1’s pressure (*P_u_*) remains uniform, matching the in-cylinder pressure (*P_a_*) at every crankshaft rotation angle;
- The gas pressure behind the top ring (*P_g_*) is assumed constant at each crank rotation angle. In control Volume 3, gas pressure (*P_l_*) is uniform and set equal to crankcase pressure, considered equivalent to atmospheric pressure (*p_atm_*) for every crank rotation angle;
- In Furuhama and Tada’s [42] study, gas temperatures in various control volumes were found to closely match the gas in-cylinder temperature. Consequently, for each crank rotation angle, we take *T_u_ = T_g_ = T_l_ = T_a_*;
- Cross-sectional areas A_1_ and A_3_ are assumed to remain constant as the piston moves upward from BDC to TDC during the compression stroke (**Case 1**). The constant values of *A*_1_ and *A*_3_ change due to the piston’s reverse motion. Likewise, they are also assumed to remain constant as the piston moves downward from TDC to BDC during the power stroke (**Case 2**).

**Figure 2:**
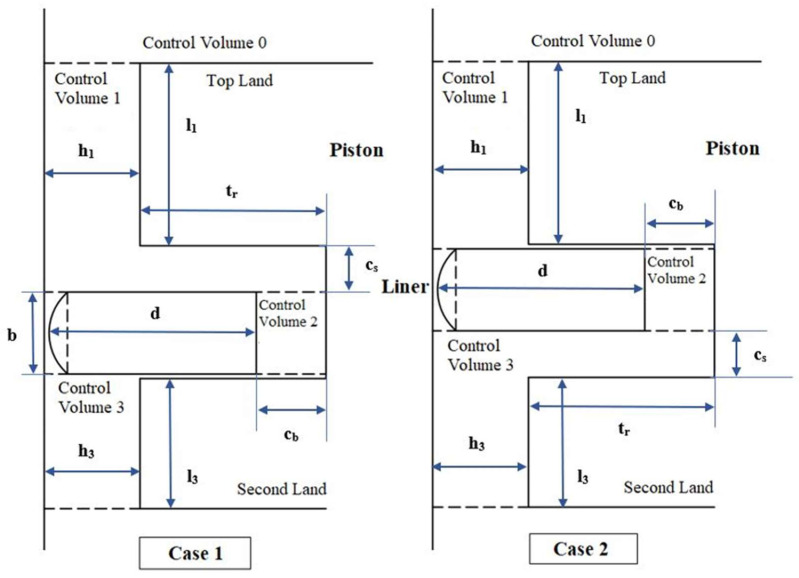
Geometric specifications of the gas blow-by control volume model.

In Figure 2, control volumes around the top ring are shown, assuming isothermal conditions at 40 °C. As can be seen, the control volumes consist of three regions with the following corresponding geometrical parameters:

- l_1_ is the top land height and l_3_ is the second land height;
- t_r_ is the piston groove radial length;
- c_b_ is the back clearance between the piston and the ring;
- c_s_ is the top clearance between the piston and the ring.

The back gas pressure is calculated using the Runge–Kutta 4th Order Method (RK4) with a fixed step size of 0.001 in an iterative process. The mass flow rates between these volumes are computed for various initial pressures, including the mass flow from control volumes 1 to 2 at a given crank angle, as follows:

(1) $${\dot{m}}_{in}={\dot{m}}_{12}=\frac{A_{1}\;{h_{1}}^{2}}{24\;l_{1}\;\eta_{g}\;R\;{Τ}_{g}}({P_{u}}^{2}-{P_{g}}^{2})$$

where *A*_1_ is the area of the control volume 1, *h*_1_ is the distance between the liner and the piston top land, *n_g_* is the dynamic viscosity of a binary gas mixture and *R* is the ideal gas constant 8.314 J⋅K^−1^⋅mol^−1^. Similarly, the mass flow from control volumes 2 to 3 is formulated as follows:

(2) $${\dot{m}}_{out}={\dot{m}}_{23}=\frac{A_{3}\;{h_{3}}^{2}}{24\;l_{3}\;\eta_{g}\;R\;{Τ}_{g}}({P_{g}}^{2}-{P_{l}}^{2})$$

By substituting Equations (1) and (2) into the equation, we derive

(3) $$\frac{{dP}_{g}}{d\varphi }=\frac{R\;{Τ}_{g}}{V_{g}\;\omega }\;\left({\dot{m}}_{in}-{\dot{m}}_{out}\right)$$

A non-linear, first-order ordinary differential equation is obtained as follows:

(4) $$\frac{{dP}_{g}(\varphi )}{d\varphi }=\frac{1}{V_{g}\;\omega \;24\;\eta_{g}}\left[\frac{A_{1}\;{h_{1}}^{2}\;\left(\;{P_{u}}^{2}-{P_{g}}^{2}\left(\varphi \right)\right)}{l_{1}}-\frac{A_{3}\;{h_{3}}^{2}\;\left({P_{g}}^{2}\left(\varphi \right)-{P_{l}}^{2}\right)}{l_{3}}\right]\;,\;\;\;\;\;\;P_{g}\left(\varphi_{0}\right)=P_{g_{0}}$$

**Figure 3:**
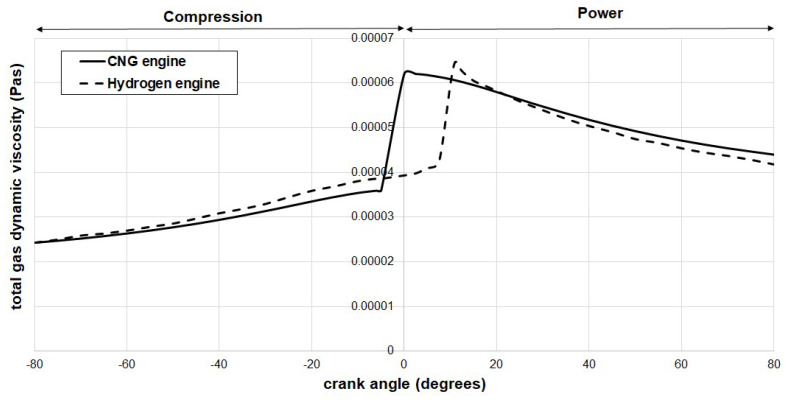
Variation of total gas dynamic viscosity for the hydrogen and the CNG engine at 1500 rpm.

Figure 3 illustrates the total dynamic viscosity of the blow-by gases for hydrogen and CNG fuels, respectively. The mathematical formulation of the gas mass flow rate from one control volume to the next also takes into account the *η_g_* coefficient, which signifies the dynamic viscosity of the blow-by gases. This coefficient is highly dependent on in-cylinder gas temperatures. At a specific crank angle, and consequently a fixed in-cylinder gas temperature, we make the assumption that the dynamic viscosity of the blow-by gases within control volumes 1, 2, and 3 is equivalent to the dynamic viscosity of the gases within the cylinder or control volume 0. In-cylinder temperatures are obtained from Elkelawy et al. [43] for hydrogen fuel, while for CNG, information from Semin et al. [44] is used as a reference.

To enhance the accuracy of numerical results, a thermal model is proposed for calculating the dynamic viscosity of a fuel–air binary mixture. This model is based on an effective method introduced by Davidson [45]. According to Davidson’s method, the fluidity, representing a fluid’s ability to transport momentum in a binary gas mixture of components 1 and 2, is formulated as follows:

(5) $$f_{1,2}=\frac{{y_{1}}^{2}}{\eta_{1}}+2\frac{y_{1}\;y_{2}}{\sqrt{\eta_{1}}\;\sqrt{\eta_{2}}}{E_{1,2}}^{A}+\frac{{y_{2}}^{2}}{\eta_{2}}$$

Here, *y_i_* is the momentum fraction of a component with molecular weight *M_i_* in the gas mixture, and it is calculated as follows:

(6) $$y_{i}=\frac{x_{i}\;\sqrt{M_{i}}}{\sum_{i}\left(x_{i}\;\sqrt{M_{i}}\right)}$$

To use Equations (5) and (6), we need to know the air–fuel equivalence ratio *λ_eq_* in advance. The computational fluid dynamic analysis for hydrogen assumes an air–fuel equivalence ratio *λ_eq_* of 0.52 [43]. While there are no data regarding the *λ_eq_* ratio for the natural gas computational fluid dynamic analysis, we assume it is also *λ_eq_* = 0.52. This assumption holds because methane, which constitutes a significant portion of compressed natural gas, is considered. Consequently, for Equations (5) and (6), the parameter $\Phi =\frac{1}{\lambda_{eq}}$ is defined as [46]:

(7) $$\Phi =\frac{fuel-to-air\;ratio}{stoichiometric\;fuel-to-air\;ratio}=\frac{m_{fuel}/m_{air}}{{\left(m_{fuel}/m_{air}\right)}_{st}}=\frac{n_{fuel}/n_{air}}{{\left(n_{fuel}/n_{air}\right)}_{st}}$$

To establish the stoichiometric fuel-to-air ratio, stoichiometric combustion reactions are essential;

(8) $$2H_{2}+1\left(O_{2}+3.76{Ν}_{2}\right)\to 2H_{2}O+3.76{Ν}_{2}$$

(9) $${CH}_{4}+2\left(O_{2}+3.76{Ν}_{2}\right)\to {CO}_{2}+2H_{2}O+7.52{Ν}_{2}$$

To implement Equation (5), we also need to find the scalar coefficient,

(10) $$B_{i,j}={\left({Ε}_{i,j}\right)}^{A}$$

This requires calculating the mean efficiency with which momentum transfers between components 1 and 2 of a binary mixture, where the empirical constant *A* is assumed to be 164 for most mixtures,

(11) $${Ε}_{1,2}=\frac{y_{1}\;{Ε}_{12}+y_{2}\;{Ε}_{21}}{y_{1}+y_{2}}$$

Finally, the dynamic viscosity of a binary gas mixture, consisting of components 1 and 2, is calculated as the reciprocal of the fluidity;

(12) $$\eta_{1,2}=\frac{1}{f_{1,2}}$$

### 2.2. Quasi-Static Piston Ring Lubrication Model

The tribological ring model, as presented in Zavos and Nikolakopoulos [18], is summarized here. The CFD model was constructed in Ansys Mechanical through Ansys Programming Design Language (APDL), which allows for full parameterization of the model input parameters and customized post-processing. Specifically, the model includes ring balance, lubricant properties, contact tribology, and gas blow-by. Hydrodynamic pressure distribution was calculated using the Navier–Stokes equations with the Finite-Volume Method (FVM), and boundary friction was assessed using the Greenwood–Tripp (GT) elastic contact model [45]. Simultaneously, the gas blow-by model was also used to approximate real gas pressure conditions. Hence, ring friction and power losses are presented and compared for various fuel IC engines. The goal of this approach was to enable the discernment of the complex interplay between blow-by, fuel, and their impact on piston ring friction, power losses, and resulting ring strength. Additionally, the CFD model can be transferred to a GUI tool developed in Python for simulating different operating conditions and to feed the structural piston ring model in ANSYS Discovery.

This model examines film thickness at the top ring–liner interface, including boundary-mixed and hydrodynamic lubrication conditions. To calculate the hydrodynamic pressure distribution, the Navier–Stokes equations are solved. The equations representing continuity and the conservation of momentum are presented below [47]:

(13) $$\frac{D\rho }{Dt}+\rho \nabla \vec{V}=0$$

(14) $$\rho \frac{D\vec{V}}{Dt}=-\nabla p+\nabla \cdot \left(\bar{\tau_{ij}}\right)+\vec{F}$$

where $\vec{V}$ is the fluid velocity vector, *p* is the pressure, *ρ* is the oil density, $\vec{F}$ is the external body forces and $\bar{\tau_{ij}}$ denotes the stress tensor, which is expressed as:

(15) $$\bar{\tau_{ij}}=\mu \left(\frac{\partial U_{i}}{\partial x_{j}}+\frac{\partial U_{j}}{\partial x_{i}}-\frac{2}{3}\delta_{ij}\nabla \cdot \vec{V}\right)$$

in which *μ* represents the lubricant viscosity, while the second term on the right-side accounts for the impact of volume dilation. In reference to cavitation, it is essential to consider the multiphase flow in this area. This involves solving multiphase flow with the Navier–Stokes equations and vapor transport [17]. However, it is important to note that in engine bearings, the impact of cavitation on load-bearing capacity and viscous friction was disregarded owing to its heightened significance.

The effective lubricant viscosity is determined using the Houpert expression [48]:

(16) $$\mu =\mu_{o}exp\left\{\left(ln\mu_{ο}+9.67\right)\left[{\left(1+\frac{p_{h}-p_{atm}}{1.98\times 10^{8}}\right)}^{Z_{o}}-1\right]\right\}$$

where the parameter *Z_o_* is presented in Ref. [49]. It is worth noting that both engines are operated at 1500 rpm and a temperature of 40 °C, which corresponds to the cold conditions specified by the New European Driving Cycle (NEDC).

In scenarios involving mixed lubrication conditions, the load of asperities and contact area are predicted using the stochastic Greenwood–Tripp (GT) model [50]:

(17) $$W_{c}=\frac{16\sqrt{2}}{15}\pi {\left(\zeta \kappa \sigma_{rms}\right)}^{2}\sqrt{\frac{\sigma_{rms}}{\kappa }}E^{\prime }AF_{5/2}\left(\lambda \right)$$

(18) $$A_{c}=\pi^{2}{\left(\zeta \kappa \sigma_{rms}\right)}^{2}AF_{2}(\lambda )\;$$

where$\;\sigma_{rms}$ is the rms surface roughness, κ is the asperity density, ζ is the asperity curvature, and the probability distribution of asperity heights, denoted by $F_{5/2}\left(\lambda \right)$ and $F_{2}\left(\lambda \right)$, is assessed using a fifth-order polynomial curve [21];

(19) $$F_{5/2}\left(\lambda \right)=\left\{\begin{array}{cc} -0.0046\lambda^{5}+0.0574\lambda^{4}-0.2958\lambda^{3}+0.7844\lambda^{2}-1.0776\lambda +0.6167, & \lambda \leq 2.224\\ \;\;0, & \lambda >2.224 \end{array}\right.$$

(20) $$F_{2}\left(\lambda \right)=\left\{\begin{array}{cc} -0.0018\lambda^{5}+0.0281\lambda^{4}-0.1728\lambda^{3}+0.5258\lambda^{2}-0.8043\lambda +0.5003, & \lambda \leq 2.295\\ \;\;0, & \lambda >2.295 \end{array}\right.$$

**Figure 4:**
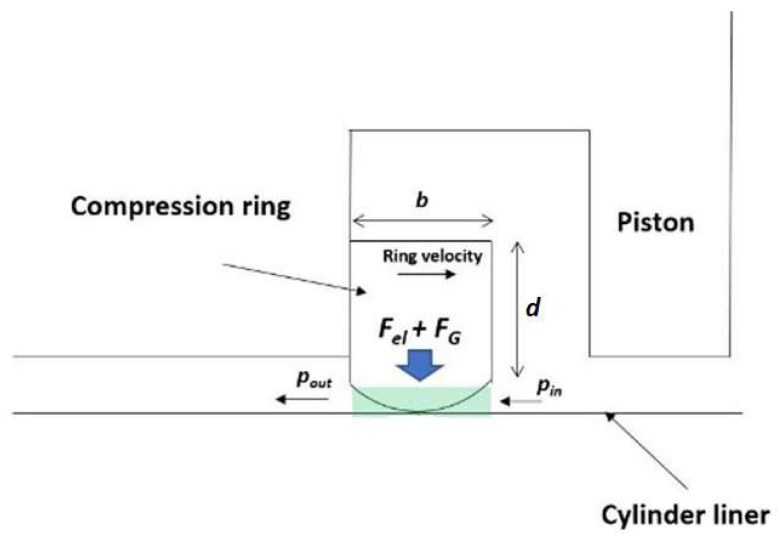
Applied forces and flow conditions in the ring–liner contact.

Figure 4 shows the top ring–liner conjunction in the piston groove, showing primary forces and flow conditions. The CFD simulation considers in-cylinder and atmospheric outlet pressures, defining boundary flow conditions as follows:

(21) $$\begin{array}{c} p\left(x,\;b/2\right)=\left\{\begin{array}{c} p_{in}=p_{\;c}\;(BDC\to TDC)\\ p_{out}=p_{\;c}\;(TDC\to BDC) \end{array}\right.\\ \;p\left(x,\;-b/2\right)=\left\{\begin{array}{c} p_{out}=p_{a}\;(BDC\to TDC)\\ p_{in}=p_{a}\;(TDC\to BDC) \end{array}\right. \end{array}$$

Here, the CFD code used the Semi-implicit Method for Pressure-linked Equations (SIMPLE) algorithm, utilizing a total of 5000 quadrilateral volumes. Grid sensitivity tests were conducted to enhance precision. Further comprehensive information on the CFD model can be found in [18].

The gas blow-by force is calculated as:

(22) $$F_{G}\left(\varphi \right)=2\;\pi \;b\;r_{0}\;P_{g}(\varphi )$$

and the tension force is:

(23) $$F_{el}=p_{el}\;b\;r_{0}$$

where $p_{el}=\frac{gE_{r}I_{r}}{3\pi b{\left(r_{0}\right)}^{4}}$ and the top ring cross-section is $I_{r}=\frac{bd^{3}}{12}$.

The ring profile reciprocates along the cylinder liner in relation to the piston sliding velocity, which is assumed as [49]:

(24) $$U_{p}\left(\varphi \right)=r\;\omega \;\left(sin\varphi +\frac{\lambda_{CR}}{2}sin2\varphi \right)$$

where *r* is the crank-pin radius, *ω* is the rotational engine speed, *φ* is the crank angle and *λ_CR_* is the control ratio. 

The film thickness can be effectively approximated as

(25) $$h(x,t)=h_{min}\left(t\right)+h_{s}(x)$$

Here, $h_{min}\left(t\right)$ represents the minimum film thickness, while $\;h_{s}\left(x\right)$ characterizes the ideal parabolic shape of the ring. It is worth noting that the cylinder liner is modeled with a circular shape, although this simplification may not mimic real-world conditions. However, this approach is specifically chosen to align with the research focus on lubrication mechanisms during contact under both CNG and hydrogen fuel conditions.

For each crank angle, the external forces are computed, including the gas blow-by force and the elastic force. Under the hydrodynamic regime of lubrication, the ring’s load capacity is defined as:

(26) $$W_{h}=2\;\pi \;r_{0}\int_{-b/2}^{b/2}p_{h}dxdy$$

Furthermore, utilizing the comprehensive GT model in conjunction with CFD simulations, the asperities contact load and initial minimum lubricant film thickness are determined. To assess the appropriateness of the initial minimum film value for the given crank angle, which is pivotal for the aforementioned calculations, a second convergence criterion is introduced into the CFD code based on the quasi-static equilibrium of radial applied forces, as expressed below:

(27) $${Error}_{load}=\left|\frac{\left(\;F_{g}\;(\varphi )+F_{el}\;\right)-\left(\;W_{h}\;(\varphi )+W_{c}\;(\varphi )\;\right)}{\left(\;F_{g}\;(\varphi )+F_{el}\;\right)}\right|\leq 10^{-3}$$

If this criterion is met at a given crank angle, the code no longer updates the minimum film thickness, and the last calculated hydrodynamic pressure is utilized along with the last minimum film thickness. However, if this criterion is not met, the minimum film is updated using the following equation:

(28) $$h_{min}^{new}=(1+\delta \frac{F\;(\varphi )-W\;(\varphi )}{max\;\left\{\;F\;(\varphi ),\;\;\;W\;(\varphi )\;\right\}})h_{min}^{old}$$

where δ=0.03 [18]. This is used to achieve faster load convergence with good numerical stability. Once the criteria for a specific crank angle and a particular minimum film are satisfied, the analysis proceeds to demonstrate the total generated ring friction and total power losses.

In conclusion, the total friction and power dissipation within the ring–liner gap are determined as follows:

(29) $$f_{tot}=f_{fl}+f_{b}$$

(30) $$P_{tot}=f_{tot}\;U_{p}\left(\varphi \right)$$

where the viscous friction is given as

(31) $$f_{fl}=\left|\pm \frac{h}{2}\nabla p-\Delta \vec{V}\frac{\mu }{h}\right|\left(A-A_{c}\right)\;$$

while the boundary friction is

(32) $$f_{b}=\tau_{o}A_{c}+\mu_{asp}W_{c}$$

in which $\tau_{o}\;$ is the non-Newtonian Eyring shear stress, holding a constant value of 2 MPa, while $\mu_{asp}\;$ represents the boundary shear strength of contact asperities. It is well known that the boundary coefficient can exhibit variations owing to factors such as wear and lubricant properties [21]. For this analysis, we have assumed $\mu_{asp}\;$ to be 0.17, a value applicable to surfaces with a layer of ferrous oxide [51]. 

### 2.3. Piston Ring Strength Analysis and Solution Methodology

**Figure 5:**
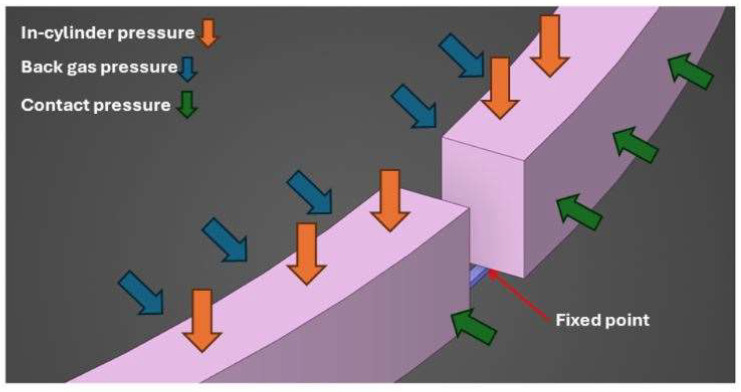
Boundary conditions on the 3D piston ring FEM model.

For the ring strength analysis, a finite element 3D model was developed using Ansys Discovery 2023 R2. The top ring, fabricated from a standard steel, was considered, with negligible contact friction assumed for other contacting regions, including groove land–ring contact and gudgeon piston bore contact. Figure 5 shows all boundary conditions applied to the top compression ring.

Despite cyclic force variations, the maximum value was utilized to assess the optimal design stress and strain under periodic loading. Grid sensitivity tests were conducted to determine the appropriate element mesh size and enhance simulation calculation accuracy. Table 1 outlines the results of the grid sensitivity tests for key performance parameters such as Von Mises stress and total deformation. Following convergence tests, an element size of 0.5 mm was deemed suitable for the intended calculations. This trend is the same as that reported by Mishra et al. [29] for a thick top compression ring under different operating conditions.

**Figure 6:**
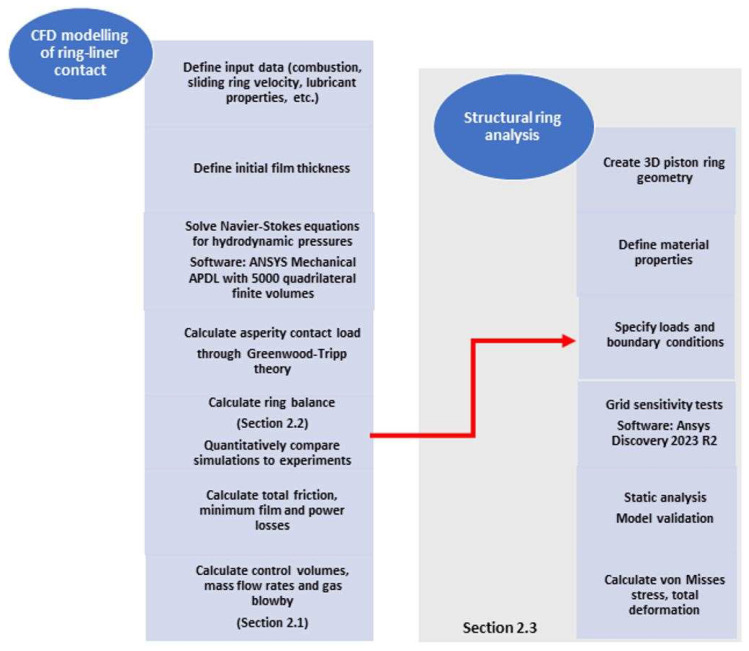
Workflow of the CFD and FEM piston ring simulations.

The proposed simulation models are integrated to encompass both the tribological and structural behaviors of the top compression ring. The workflow depicted in Figure 6 illustrates the process for conducting both Computational Fluid Dynamics (CFD) analysis and Finite Element Method (FEM) simulations of the piston ring.

## 3. Validation

The tribological model employed for mixed lubrication conditions was validated against experimental data obtained by Zavos and Nikolakopoulos [52]. Utilizing the strain gauge method, the experimental friction results from a four-stroke motor engine were juxtaposed with the Computational Fluid Dynamics (CFD) outcomes. The analysis involved modeling a thin, newly-designed top compression ring, with its axial profile represented by a 6th order polynomial curve in the simulation. The input conditions utilized for validation are outlined in Table 2.

**Figure 7:**
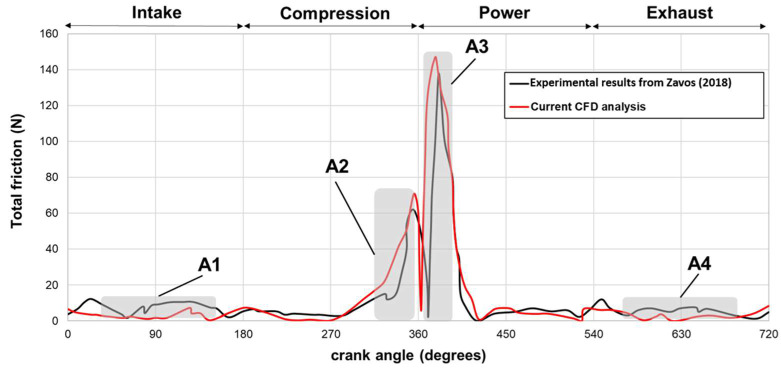
Validation results. Comparison between experimental friction from Zavos and Nikolakopoulos [52] and CFD results from the current analysis.

As illustrated in Figure 7, the maximum CFD ring friction exhibited favorable agreement during the transition from compression to power strokes through Top Dead Center (TDC) reversal. However, discrepancies became more pronounced as the ring reached the midstroke position, attributed to complex ring motion influenced by high inertial forces and elastodynamic behavior. This complexity arises from both out-of-plane and in-plane ring motions within the piston groove. The standard deviations at each engine stroke are depicted in the figure, revealing four distinct regions for evaluating relative deviations. Region A1, corresponding to the intake stage, displayed a substantial discrepancy, with the average CFD ring friction significantly lower (52%) than the measured values. In regions A2 and A3, spanning from compression to power strokes at the TDC zone (crank angles of 280–420°), the difference amounted to 23%. Furthermore, in region A4, during the exhaust stage, the average deviation was 30%. This observation aligns with findings reported by Baker et al. [53] when comparing static and dynamic models.

**Figure 8:**
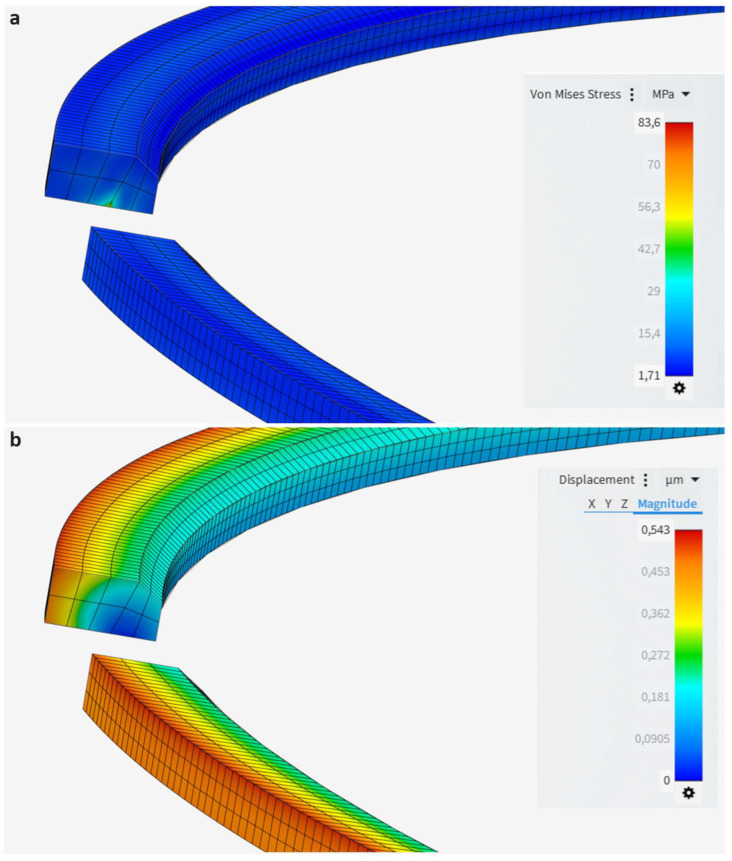
Validation results. (**a**) Equivalent Von Mises stress and (**b**) total deformation of the top piston ring from the current analysis using data from Mishra et al. [29].

For the strength analysis, Mishra et al. [29] employed piston ring pack elastodynamics to explore the impacts of the piston crown and coatings on piston and ring pack strength. Mishra provided detailed information on geometric and meshing conditions, along with operating parameters such as combustion and contact forces, facilitating the validation process. Their study revealed that the top piston ring exhibited a maximum Von Mises stress of 92.64 MPa, with a total deformation of 0.62 μm. Figure 8 illustrates the Von Mises stresses and total deformation of the top piston ring using Ansys Discovery 2023 R2. The FEM simulation in this study achieved good agreement with the corresponding values published by Mishra. Specifically, the standard differences were 9.7% for the maximum Von Mises stress and 12.9% for the total deformation, respectively.

## 4. Results and Discussion

### 4.1. Performance Characteristics of CNG and Hydrogen-Fueled Engines

The power loss investigations were conducted on two distinct engine designs, meticulously selected to facilitate comprehensive comparisons. These comparisons extend beyond the different fuel types to encompass the intricacies of their respective piston geometries in gas blow-by. The lighter hydrogen-fueled SI engine was sourced from Elkelawy et al. [43], while the heavier CNG-fueled SI engine’s specifications were derived from Semin et al. [44]. Notably, both engines share nearly identical volume displacement, with the hydrogen engine being lighter than the CNG test ship engine. Additionally, both engines employ Port Fuel Injection (PFI) delivery systems. Table 3 provides a concise overview of the engine details.

For consistency and relevance to the specific engines under investigation, the ring face width was set at a maximum of 2.5 mm, aligning with recommendations found in previous studies [54] and ISO 6622-2:2013 [36]. The top compression rings utilized in this study are constructed from standard steel, and roughness parameters were chosen in accordance with similar research [17,54], where thick high-performance compression rings were employed. Table 4 provides the input data used in ring tribology analyses for both IC engines.

### 4.2. Comparative Analysis of Ring Friction and Energy Losses 

**Figure 9:**
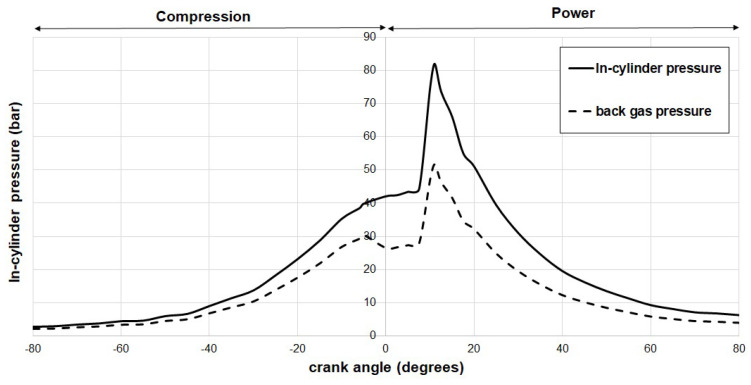
Variation of in-cylinder pressure and top compression ring back pressure in hydrogen IC engine at 1500 rpm.

**Figure 10:**
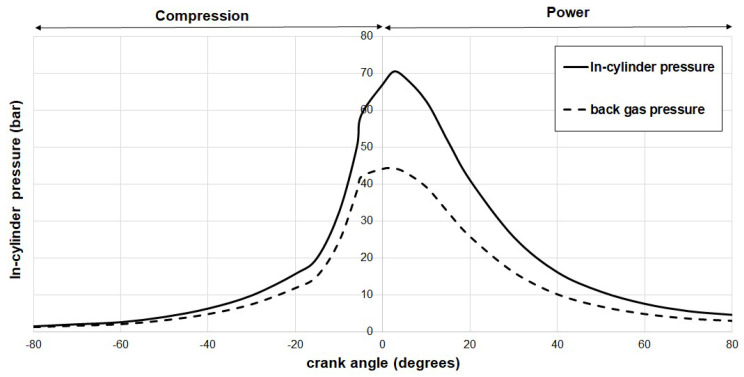
Variation of in-cylinder pressure and top compression ring back pressure in CNG IC engine at 1500 rpm.

Figure 9 and Figure 10 illustrate the calculated back gas pressure within the top compression ring for hydrogen and CNG (compressed natural gas)-fueled engines, respectively. To obtain these results, a numerical method was employed on a computing platform, with computations conducted for crankshaft angles ranging from −80° to 80° in 5° increments, while maintaining an engine speed of 1500 rpm. As previously mentioned, the in-cylinder pressure data used for these calculations were sourced from Elkelawy et al. [43] and Semin et al. [44].

In both hydrogen- and CNG-fueled engines, the points of maximum pressure occurrence were observed at 5 degrees and 10 degrees past the Top Dead Center (TDC), respectively. Under representative stoichiometric conditions, it was observed that the hydrogen engine exhibited a maximum back pressure 16% higher than that of the CNG engine. This discrepancy can be attributed to the higher in-cylinder pressures and reduced cross-sectional areas around the top ring associated with hydrogen engine pistons. Conversely, the CNG engine, with its piston geometry and lower in-cylinder pressures, demonstrated lower back pressures within the same piston ring. These findings bear implications for the sealing behavior of the top ring in both engine types. To emphasize the similarities and potential differences between the two fuels, the gas pressure values were compiled and are presented in Table 5. This table includes gas pressures behind the first compression ring for various crank angles, alongside the corresponding percentage of pressure drop.

**Figure 11:**
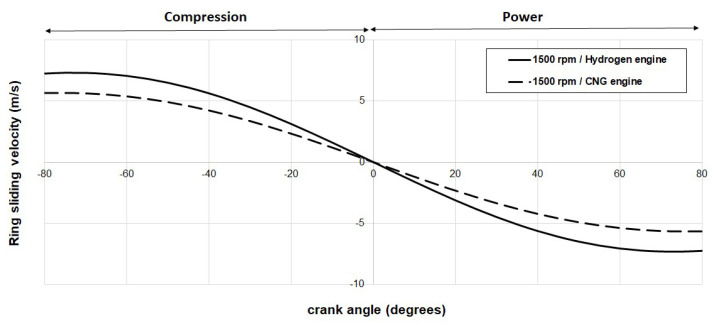
Predicted ring sliding velocity for a hydrogen engine (solid line) and a CNG engine (dashed line) at 1500 rpm.

Figure 11 provides the calculated piston velocity profiles for both internal combustion (IC) engines. A focused examination of the predicted sliding velocities is particularly encouraged for the Top Dead Center (TDC) reversal, where mixed lubrication conditions are prevalent.

**Figure 12:**
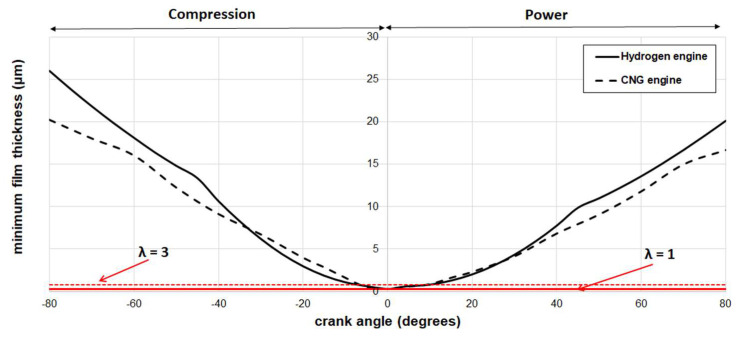
Predicted minimum film for the hydrogen engine (solid black line) and the CNG engine (dashed black line) at 1500 rpm.

Figure 12 provides a comparative analysis of the minimum film thickness in engines fueled by hydrogen and CNG, operating at 1500 rpm. Understanding contact conditions and the tribological performance of piston rings hinges on factors such as sliding velocity and lubricant temperature. In this study, while maintaining constant lubricant temperature, variations in sliding velocity (as shown in Figure 11) arise due to differences in engine type. Specifically, at the TDC position, the minimum oil film thickness is approximately 0.27 μm, indicating boundary lubrication when λ ≤ 1, and mixed lubrication when the Stribeck oil film ratio is λ < 3. Interestingly, both fueled engines exhibit a lower minimum film thickness at the TDC region. This disparity exacerbates asperity interactions and elevates boundary friction due to limited lubricant motion in the contact region. This observation aligns with the findings of other researchers [55,56], who have reported similar trends through friction measurements and numerical predictions. Excluding the mixed lubrication region, the minimum oil film is greater for the hydrogen IC engine than its CNG counterpart. Consequently, for this hydrogen engine system, not only does the applied load on the ring increase, but the higher sliding velocity, influenced by distinct engine parameters, further exacerbates the situation. 

**Figure 13:**
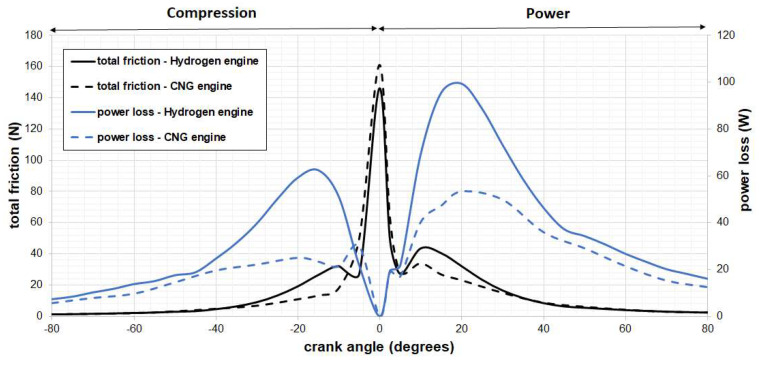
Predicted total friction and power loss for the hydrogen engine (solid line) and the CNG engine (dashed line) at 1500 rpm.

Figure 13 illustrates the comparison of predicted total ring friction and power loss in engines fueled by hydrogen and compressed natural gas (CNG). Clearly, there is a noticeable increase in total friction at TDC, with the maximum friction occurring during the transition between the compression stroke and the power stroke. This can be attributed to the significant back gas load on the top ring, and its lower speed. Additionally, as sliding velocity increases, the influence of viscous shear becomes more pronounced. These observations are consistent with the findings of Zavos and Nikolakopoulos [52], which include experimental predictions from a small motorbike engine. However, it is essential to highlight that the choice of fuel and piston system directly impacts ring contact conditions through gas blow-by. In this analysis, the use of a CNG IC engine can lead to more challenging tribological conditions by extending the prevalence of mixed lubrication regimes. In contrast, a smaller hydrogen IC engine can result in higher viscous losses, ultimately increasing overall power losses. Here, we observe that the contribution of viscous friction becomes increasingly significant after TDC in the hydrogen engine system, where piston velocity is higher, and the piston is lighter than in the case of a CNG engine. The predicted frictional power losses were determined using the CFD model, which utilized the simulated friction forces between the top piston rings and the cylinder liner in both engines. In this scenario, the power losses were found to be approximately 34.7% higher for the hydrogen engine compared to the CNG case.

**Figure 14:**
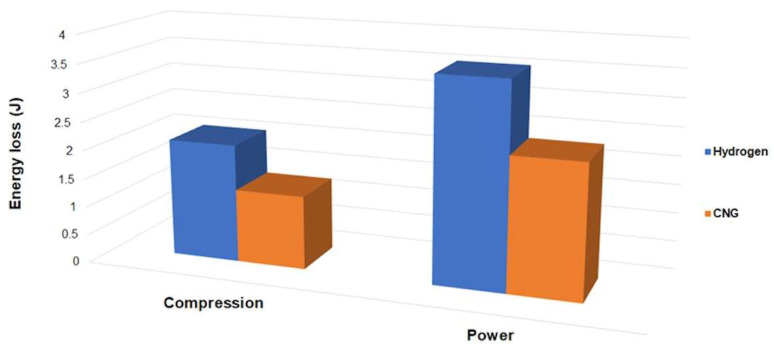
Predicted average energy losses at 1500 rpm.

Figure 14 illustrates the energy lost due to the friction of the top piston rings against the cylinder. These losses are calculated by integrating the instantaneous power losses over the entire engine cycle using the following equation:

(33) Eloss=2ω60(Ptot)avg

where *ω* represents the engine speed in rpm. The results indicate that the losses associated with the hydrogen engine are higher compared to the CNG engine, ranging from 37.9% at the compression stage to 34.5% at the power stroke. It is important to note that the current analysis is based on several assumptions, including constant temperature and rigid ring behavior. Future work on tribological modeling can enhance accuracy by incorporating additional thermodynamic details regarding heat release and transfer models for alternative fuels, as well as considering other tribological phenomena such as ring dynamics and evolving lubricant properties. These refinements hold the potential to allow more precise predictions.

### 4.3. Effect of Hydrogen Delivery Method on Top Compression Ring Performance

Hydrogen exhibits high engine efficiency and a broad flammability range, as reported in various studies [57,58]. However, utilizing hydrogen in conventional internal combustion (IC) systems presents challenges, including pre-ignition, knocking, and backfire. To address these issues, Port Fuel Injection (PFI) and Direct Injection (DI) ignition systems require meticulous adjustments to enhance thermal efficiency while managing NOx emissions [59].

In this study, the combined tribological and structural performance of the top piston ring is analyzed, considering two alternative hydrogen fuel delivery systems. As noted by Elkelawy et al. [43], the maximum in-cylinder pressure is 9.82 MPa with the Direct Injection (DI) configuration, which is 17.7% higher than that of the PFI fuel delivery system. Under these conditions, the generated energy loss is 10.1% higher for the hydrogen fuel under the DI method, suggesting that the structural integrity of the top piston ring would be more affected. Therefore, material technology plays a crucial role in improving the piston ring integrity, especially during large-scale production, covering a range from high- to ultra-high strength. 

**Figure 15:**
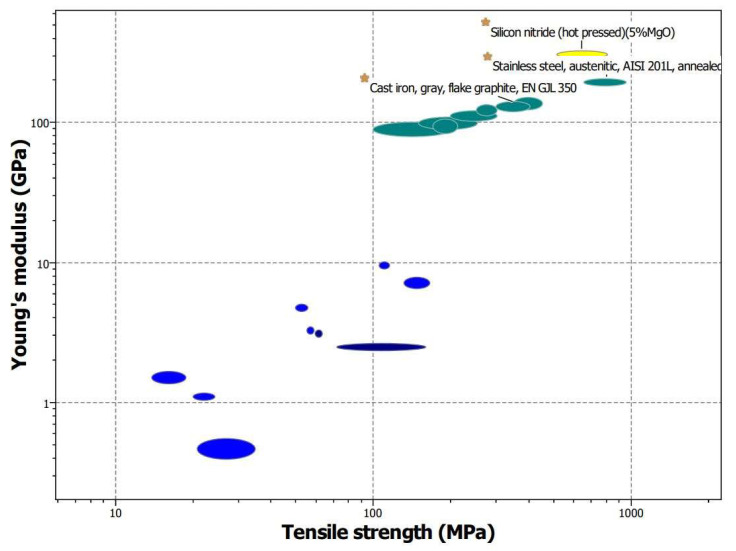
Young’s Modulus vs. total tensile strength of base materials in piston rings.

In this context, three base materials are tested and compared numerically, each offering a combination of high strength and modulus of elasticity (see Figure 15). Table 6 presents the main mechanical properties of these materials. Parametric simulations are conducted using Ansys Discovery 2023 R2, with material data obtained from Granta Selector 2023 R2 [60]. It is important to note that this study focuses on investigating the base ring materials, with an in-depth study of coatings proposed for future research. Additionally, future work may include incorporating thermal effects into FEM predictions to analyze the interconnected impacts of coatings and fuel on frictional performance and durability.

**Figure 16:**
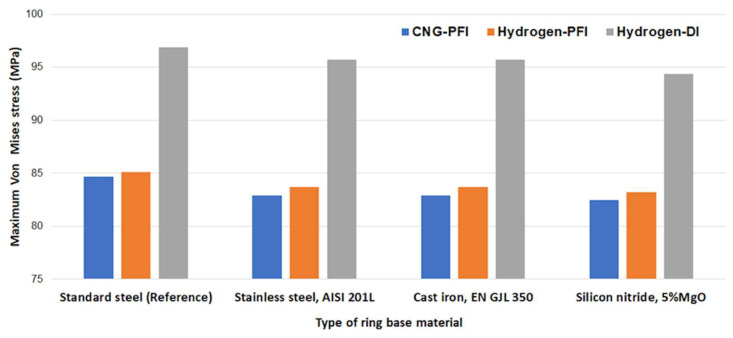
Predicted maximum Von Mises stress under different ring materials and engine conditions at 1500 rpm.

The corresponding predicted maximum Von Mises stress under different cylinder operating conditions is depicted in Figure 16. The Von Mises criteria are crucial for understanding the failure mode approach, as they are based on distortion energy or shear strain energy [61]. The Direct Injection (DI) configuration exhibits the highest Von Mises stress at the Top Dead Center (TDC). This is attributed to the higher combustion pressure within the DI delivery fuel system in the hydrogen engine compared to both the hydrogen–PFI engine and the CNG–PFI engine. As shown in Figure 16, the maximum stress is 12.1% higher for the hydrogen fuel under the DI method compared to the hydrogen–PFI and CNG–PFI configurations. However, the difference is limited to 2.3% between the hydrogen–PFI and the CNG–PFI engines, respectively. Furthermore, the silicon nitride piston ring demonstrates superior strength compared to others due to its favorable material properties. The combination of base material properties and coating layers can aid in determining the best design for the piston ring, especially in scenarios with severely reduced lubricant film thickness. Further investigations are required to fully understand this behavior.

## 5. Conclusions

This study investigates the impacts of alternative fuels, specifically hydrogen and CNG, on top piston ring behavior within internal combustion (IC) engines. Two types of IC engines were analyzed, considering flow conditions derived from in-cylinder pressures and piston geometry. Gas blow-by was forecasted using an iterative approach, and a CFD code was created for predicting ring friction and energy losses. Additionally, an interactive FEM ring model was built for both engines with varying materials to predict structural performance. This comprehensive methodology was subjected to verification against experimental and numerical references, resulting in a commendable degree of agreement. In light of these investigations, the following conclusions can be derived:

- The gas blow-by predictions reveal the significant impact of engine parameters, notably piston geometry, on hydrogen- and CNG-fueled IC engines. The lighter hydrogen-fueled engine exhibited increased back gas pressure at the Top Dead Center (TDC) region due to its narrower ring gaps and higher in-cylinder pressure. In contrast, the heavier CNG IC engine demonstrated comparatively lower back gas pressures. Consequently, the hydrogen IC engine leads to higher viscous losses, resulting in a 34.7% increase in overall power losses compared to the CNG case. The similar idle speed, ignition system, and use of Port Fuel Injection (PFI) in both engines facilitate the extraction of in-cylinder pressure curves for prototype numerical investigations. Both in-cylinder pressure curves peak in the 70–80 bar range around Top Dead Center (TDC), validating their comparability;
- The analysis also highlights the importance of considering fuel delivery systems in frictional loss predictions using hydrogen. Particularly, the higher in-cylinder pressure using Direct Injection (DI) showed greater ring energy losses, by approximately 10.1%, compared to Port Fuel Injection (PFI). At the same time, regarding the choice of base ring material, the maximum Von Mises stress is 12.1% higher for the hydrogen fuel with the DI method compared to the hydrogen–PFI and CNG–PFI cases using the silicon nitride top ring, due to its superior strength compared to standard steel, stainless steel (AISI 201L), and cast iron (EN GJL 350).

While this analysis has been comprehensive, it retains certain assumptions, particularly concerning the thermodynamics of alternative fuels and the tribological ring model. The pursuit of a holistic thermodynamics and tribodynamics analysis holds the promise of furnishing a more exhaustive understanding of the most pertinent operating conditions. This endeavor is part of our ongoing research, as we continue to refine and deepen our insights into the intricate interplay of fuels and engine systems.

## Figures and Tables

**Table 1:** Grid sensitivity tests.

Test	Element Size (mm)	Number of Elements	Von-Misses Stress (MPa)	Total Deformation (μm)
1	1	1654	31.4	0.21
2	0.8	1984	34.5	0.218
3	0.6	4900	39.1	0.22
4	0.5	9508	83.2	0.23
5	0.4	13,972	82.7	0.25

**Table 2:** Validation input parameters.

Parameter	Symbol	Value
Ring Young’s modulus of elasticity (GPa)	*E_r_*	279
Ring Poisson’s ratio	*ν_l_*	0.21
Ring face width (mm)	*b*	0.5
Ring radial width (mm)	*d*	2
Ring end gap (mm)	*g*	0.2
Ring roughness (μm)	*σ_r_*	0.71
Roughness parameter (-)	*ζκσ_rms_*	0.048
Measure of asperity gradient (-)	*σ_rms_/* *κ*	0.116
Liner Young’s modulus of elasticity (GPa)	*E_l_*	70
Liner Poisson’s ratio	*ν_l_*	0.33
Liner roughness (μm)	*σ_l_*	0.15

**Table 3:** Technical data of the IC engines.

Parameter	Hydrogen Engine	CNG Engine
Engine type	four stroke	four stroke
Volume displacement (cc)	415	406
Compression ratio	14	14.5
Bore (mm)	76	86
Stroke (mm)	88	70
Crank-pin radius (mm)	44	35
Connecting rod length (mm)	131	138.1
Idle speed (rpm)	1500	1500
Ignition system	Spark	Spark
Lubricant type	SAE10W40	SAE10W40
Lubricant density (kg.m^−3^)	850	850
Lubricant viscosity at the inlet (Pa.s)	0.07	0.07
Viscosity–temperature coefficient (K^−1^)	4 × 10^−2^	4 × 10^−2^
Viscosity–pressure coefficient (Pa^−1^)	1 × 10^−8^	1 × 10^−8^
Lubricant temperature (°C)	40	40

**Table 4:** The input data used in ring tribology analyses for both IC engines.

Parameter	Symbol	Value
Ring Young’s modulus (GPa)	*E_r_*	209
Ring Poisson’s ratio	*ν_l_*	0.3
Ring face width (mm)	*b*	2.5
Ring radial width (mm)	*d*	3.5
Ring curvature (μm)	*c*	9.55
Ring end gap (mm)	*g*	10
Ring roughness (μm)	*σ_r_*	0.25
Roughness parameter (-)	*ζκσ_rms_*	0.04
Measure of asperity gradient (-)	*σ_rms_/* *κ*	0.0015
Liner Young’s modulus (GPa)	*E_l_*	70
Liner Poisson’s ratio	*ν_l_*	0.33
Liner roughness (μm)	*σ_l_*	0.1

**Table 5:** The predicted results of gas blow-by in hydrogen and CNG IC engines.

Stroke	Hydrogen IC Engine	Pressure Drop	CNG IC Engine	Pressure Drop
**Compression**	*P_a,min_* @−80° [bar]	20%	*P_a,min_* @ −80° [bar]	13%
	2.7		1.5	
	*P_g_*_,min_ @ −80° [bar]		*P_g,min_* @ −80° [bar]	
	2.1		1.3	
**Power**	*P_a,max_* @ 10° [bar]	37%	*P_a,max_* @ 5° [bar]	37%
	81.9		68.8	
	*P_g,max_* @ 10° [bar]		*P_g,max_* @ 5 ° [bar]	
	51.5		43.3	
	*P_a,min_* @ 80° [bar]	36%	*P_a,min_* @ 80 ° [bar]	35%
	6.2		4.6	
	*P_g,min_* @ 80° [bar]		*P_g,min_* @ 80° [bar]	
	3.9		3	

**Table 6:** Mechanical properties of the tested materials.

Material	Standard Steel (Reference)	Stainless Steel, AISI 201L	Cast Iron, EN GJL 350	Silicon Nitride, 5%MgO
Young’s modulus (GPa)	209	197	94.5	310
Poisson’s ratio (-)	0.3	0.27	0.27	0.26
Density (kg/m^3^)	7850	7800	7300	3150
Bulk modulus (GPa)	166	142	70	219
Shear modulus (GPa)	76.9	77.5	37	122
Tensile Yield Strength (MPa)	250	310	124	644
Tensile Ultimate Strength (MPa)	460	788	188	644

## Data Availability

The data presented in this study are available on request from the corresponding author.

## Nomenclature

*A*	apparent contact area	m^2^
*A_c_*	asperities contact area	m^2^
*A*_1_, *A*_2_, *A*_3_	cross-sectional areas of control volumes 1,2 and 3	m^2^
*t*	flat-faced ring radial thickness	m
*b*	ring face width	m
*c*	ring curvature	m
*c_b_*	back clearance between the piston and the ring	m
*c_s_*	top clearance between the piston and the ring	m
*d*	barrel-faced ring radial thickness	m
*E_loss_*	energy loss	J
*E_l_*	Young’s modulus of elasticity for liner	N/m^2^
*E_r_*	Young’s modulus of elasticity for ring	N/m^2^
*E*′	equivalent modulus of elasticity	N/m^2^
*F*_5/2_, *F*_2_	statistical functions from GT approach	(-)
*f_tot_*	total friction	N
*f_fl_*	viscous friction	N
*f_b_*	boundary friction	N
*F_G_*	combustion gas force	N
*F_el_*	ring tension force	N
*g*	ring free end gap	m
*h*	lubricant film thickness	m
*h_s_*	ring face profile	m
*h_min_*	minimum film thickness	m
*h*_1_	distance between the liner and the piston top land	m
*h*_3_	distance between the liner and the piston second land	m
*I_r_*	second moment of ring	m^4^
*l*	connecting rod length	m
*l*_1_	top land height	m
*l*_3_	second land height	m
*m*	gas mass with regard to control volume	kg
$\dot{m}$	gas mass flow rate	kg/s
*n_g_*	dynamic viscosity of a binary gas mixture	Pa.s
*P_tot_*	total power loss	W
*P_a_*	in-cylinder gas pressure	N/m^2^
*P_l_*	pressure on the lower ring face	N/m^2^
*P_u_*	pressure on the upper ring face	N/m^2^
*P_g_*	gas pressure behind the piston ring	N/m^2^
*p_el_*	elastic pressure	N/m^2^
*p_h_*	hydrodynamic pressure	N/m^2^
*p_atm_*	atmospheric pressure	N/m^2^
*p_c_*	combustion pressure	N/m^2^
*p_in_*	inlet gas pressure	N/m^2^
*p_out_*	outlet gas pressure	N/m^2^
*R*	ideal gas constant	J⋅K^−1^⋅mol^−1^
*r*	crank-pin radius	m
*r*_0_	nominal cylinder radius	m
*t_r_*	piston groove radial length	m
*T_a_*	in-cylinder gas temperature	K
*T_av_*	average lubricant temperature	Κ
*T_u_*	gas temperature above top compression ring	K
*T_g_*	gas temperature behind the top compression ring	Κ
*T_o_*	lubricant ambient temperature	Κ
*U_p_*	piston sliding velocity	m/s
*V_a_*	combustion chamber volume	m^3^
*V_g_*	volume behind the top compression ring	m^3^
*V_l_*	volume below piston ring and the second ring groove	m^3^
*V_u_*	volume above top compression ring	m^3^
*W_h_*	hydrodynamic load	Ν
*W_c_*	asperities contact load	Ν
**Greek symbols**		
*δ*	damping coefficient	(-)
*ζκσ_rms_*	roughness parameter	(-)
*κ*	average asperity tip radius	(-)
*λ*	Stribeck oil film parameter	(-)
*μ*	lubricant dynamic viscosity	Pa.s
*μ_asp_*	coefficient parameter of boundary shear strength	Pa.s
*μ_ο_*	lubricant dynamic viscosity at atmospheric pressure and ambient temperature	Pa.s
*ν_r_*,*ν*_l_	Poisson’s ratio of ring and liner	(-)
*ρ*	lubricant density	kg.m^−3^
*σ_r_*,*σ*_l_	roughness parameter of ring and liner	μm
*σ_rms_*	RMS roughness of examined tribo pair	μm
*τ*	viscous shear stress of the lubricant film	Pa
*τ_ο_*	Eyring shear stress of the lubricant film	Pa
*φ*	crank rotation angle	Degrees
Φ	equivalence ratio (1/*λ_eq_*)	(-)
*ω*	rotational engine speed	rpm

## Abbreviations

BDC	Bottom Dead Center
CFD	Computational Fluid Dynamics
CNG	Compressed Natural Gas
DI	Direct Injection
FVM	Finite Volume Method
GT	Greenwood–Tripp
HICE	Hydrogen-fueled Internal Combustion Engines
IC	Internal Combustion
NEDC	New European Drive Cycle
PFI	Port Fuel Injection
RMS	Root Mean Square
SIMPLE	Semi-Implicit Method for Pressure Linked Equations
TDC	Top Dead Centre

## References

[1] Selleri T., Gioria R., Melas A.D., Giechaskiel B., Forloni F., Villafuerte P.M., Demuynck J., Bosteels D., Wilkes T., Simons O. (2022). Measuring Emissions from a Demonstrator Heavy-Duty Diesel Vehicle under Real-World Conditions—Moving Forward to Euro VII. Catalysts.

[2] Reitz R.D., Ogawa H., Payri R., Fansler T., Kokjohn S., Moriyoshi Y., Agarwal A., Arcoumanis D., Assanis D., Bae C. (2020). IJER editorial: The future of the internal combustion engine. Int. J. Engine Res..

[3] Onorati A., Payri R., Vaglieco B., Agarwal A., Bae C., Bruneaux G., Canakci M., Gavaises M., Günthner M., Hasse C. (2022). The role of hydrogen for future internal combustion engines. Int. J. Engine Res..

[4] Hwang J., Maharjan K., Cho H. (2023). A review of hydrogen utilization in power generation and transportation sectors: Achievements and future challenges. Int. J. Hydrogen Energy.

[5] Szamrej G., Karczewski M. (2024). Exploring Hydrogen-Enriched Fuels and the Promise of HCNG in Industrial Dual-Fuel Engines. Energies.

[6] Unich A., Bata R.M., Lyons D.W. (1993). Natural Gas: A Promising Fuel for I.C. Engines.

[7] Mathai R., Malhotra R., Subramanian K., Das L. (2012). Comparative evaluation of performance, emission, lubricant and deposit characteristics of spark ignition engine fueled with CNG and 18% hydrogen-CNG. Int. J. Hydrogen Energy.

[8] Das L. (1996). Hydrogen-oxygen reaction mechanism and its implication to hydrogen engine combustion. Int. J. Hydrogen Energy.

[9] Zavos A., Nikolakopoulos P.G. (2022). Investigation of the top compression ring power loss and energy consumption for different engine conditions. Tribol.-Mater. Surf. Interfaces.

[10] Richardson D.E. (2000). Review of Power Cylinder Friction for Diesel Engines. J. Eng. Gas Turbines Power.

[11] Holmberg K., Andersson P., Erdemir A. (2012). Global energy consumption due to friction in passenger cars. Tribol. Int..

[12] Castleman R.A. (1936). A Hydrodynamical Theory of Piston Ring Lubrication. Physics.

[13] Furuhama S. (1959). A Dynamic Theory of Piston-Ring Lubrication: 1st Report, Calculation. Bull. JSME.

[14] Akalin O., Newaz G.M. (2001). Piston Ring-Cylinder Bore Friction Modeling in Mixed Lubrication Regime: Part II—Correlation With Bench Test Data. J. Tribol..

[15] Furuhama S., Sasaki S. (1983). New Device for the Measurement of Piston Frictional Forces in Small Engines. AE Trans..

[16] Baelden C., Tian T. (2014). A Dual Grid Curved Beam Finite Element Model of Piston Rings for Improved Contact Capabilities. SAE Int. J. Engines.

[17] Shahmohamadi H., Rahmani R., Rahnejat H., Garner C.P., King P.D. (2013). Thermo-Mixed Hydrodynamics of Piston Compression Ring Conjunction. Tribol. Lett..

[18] Zavos A., Nikolakopoulos P.G. (2017). Computational fluid dynamics analysis of top compression ring in mixed lubrication. Mech. Ind..

[19] Morris N., Rahmani R., Rahnejat H., King P.D., Fitzsimons B. (2013). Tribology of piston compression ring conjunction under transient thermal mixed regime of lubrication. Tribol. Int..

[20] Gore M., Morris N., Rahmani R., Rahnejat H., King P.D., Howell-Smith S. (2017). A combined analytical-experimental investigation of friction in cylinder liner inserts under mixed and boundary regimes of lubrication. Lubr. Sci..

[21] Zavos A. (2021). Effect of Coating and Low Viscosity Oils on Piston Ring Friction under Mixed Regime of Lubrication through Analytical Modelling. Lubricants.

[22] Tung S.C., Gao H. (2003). Tribological characteristics and surface interaction between piston ring coatings and a blend of energy-conserving oils and ethanol fuels. Wear.

[23] Zhuo S., Peijun Z., Leheng Z., Xinfu X., Aimin H., Wenquan Z. (2000). Multi-layer compound coating on cast iron piston ring by multi-arc and magnetron sputtering ion compound plating technique. Surf. Coat. Technol..

[24] Posmyk A., Bakowski H. (2013). Wear Mechanism of Cast Iron Piston Ring/Aluminum Matrix Composite Cylinder Liner. Tribol. Trans..

[25] Zabala B., Igartua A., Fernández X., Priestner C., Ofner H., Knaus O., Abramczuk M., Tribotte P., Girot F., Roman E. (2017). Friction and wear of a piston ring/cylinder liner at the top dead centre: Experimental study and modelling. Tribol. Int..

[26] Wróblewski P. (2020). Technology for Obtaining Asymmetries of Stereometric Shapes of the Sealing Rings Sliding Surfaces for Selected Anti-Wear Coatings.

[27] Dziubak T., Dziubak S.D. (2022). A Study on the Effect of Inlet Air Pollution on the Engine Component Wear and Operation. Energies.

[28] Dolatabadi N., Forder M., Morris N., Rahmani R., Rahnejat H., Howell-Smith S. (2020). Influence of advanced cylinder coatings on vehicular fuel economy and emissions in piston compression ring conjunction. Appl. Energy.

[29] Mishra P.C., Roychoudhury A., Banerjee A., Saha N., Das S.R., Das A. (2023). Coated Piston Ring Pack and Cylinder Liner Elastodynamics in Correlation to Piston Subsystem Elastohydrodynamic: Through FEA Modelling. Lubricants.

[30] Chen T., Wang L., Xu J., Gao T., Qin X., Yang X., Cong Q., Jin J., Liu C. (2022). Effect of Groove Texture on Deformation and Sealing Performance of Engine Piston Ring. Machines.

[31] Gomesantunes J.M., Mikalsen R., Roskilly A.P. (2008). An investigation of hydrogen-fuelled HCCI engine performance and operation. Int. J. Hydrogen Energy.

[32] Balyts’kyi O.I., Abramek K.F., Mruzik M., Stoeck T., Osipowicz T. (2017). Evaluation of the Losses of Hydrogen-Containing Gases in the Process of Wear of Pistons of an Internal-Combustion Engine. Mater. Sci..

[33] Kindrachuk M., Volchenko D., Balitskii A., Abramek K.F., Volchenko M., Balitskii O., Skrypnyk V., Zhuravlev D., Yurchuk A., Kolesnikov V. (2021). Wear Resistance of Spark Ignition Engine Piston Rings in Hydrogen-Containing Environments. Energies.

[34] Rahmani R., Dolatabadi N., Rahnejat H. (2023). Multiphysics performance assessment of hydrogen fuelled engines. Int. J. Engine Res..

[35] Ferrarese A., Marques G., Tomanik E., Bruno R., Vatavuk J. (2010). Piston Ring Tribological Challenges on the Next Generation of Flex-fuel Engines. SAE Int. J. Engines.

[36] (2013). Internal Combustion Engines—Piston Rings—Part 2: Rectangular Rings Made of Steel.

[37] Baker C., Rahmani R., Karagiannis I., Theodossiades S., Rahnejat H., Frendt A. (2014). Effect of Compression Ring Elastodynamics Behaviour upon Blowby and Power Loss. SAE Technical Papers.

[38] Gulwadi S.D. (2000). Analysis of Tribological Performance of a Piston Ring Pack. Tribol. Trans..

[39] Ruddy B.L., Dowson D., Economou P.N. (1981). A Theoretical Analysis of the Twin-Land Type of Oil-Control Piston Ring. J. Mech. Eng. Sci..

[40] Turnbull R., Dolatabadi N., Rahmani R., Rahnejat H. (2020). An assessment of gas power leakage and frictional losses from the top compression ring of internal combustion engines. Tribol. Int..

[41] Namazian M., Heywood J.B. (1982). Flow in the Piston-Cylinder-Ring Crevices of a Spark-Ignition Engine: Effect on Hydrocarbon Emissions, Efficiency and Power.

[42] Furuhama S., Tada T. (1961). On the Flow of Gas Through the Piston-Rings: 1st Report, The Discharge Coefficient and Temperature of Leakage Gas. Bull. JSME.

[43] Elkelawy M., Bastawissi H.A.-E. (2013). Numerical Study on the Hydrogen Fueled SI Engine Combustion Optimization through a Combined Operation of DI and PFI Strategies. Energy Power Eng..

[44] Ismail A., Nugroho T. (2010). Experimental and Computational of Engine Cylinder Pressure Investigation on the Port Injection Dedicated CNG Engine Development. J. Appl. Sci..

[45] Davidson T.A. A Slmple and Accurate Method for Calculatlng Viscosity of Gaseous Mixtures I UNITED STATES DEPARTMENT OF THE INTERIOR I. https://www.osti.gov/biblio/6129940.

[46] Speight J.G. (2020). Combustion of hydrocarbons. Handbook of Industrial Hydrocarbon Processes.

[47] White F.M. (1991). Viscous Fluid Flow.

[48] Houpert L. (1985). New Results of Traction Force Calculations in Elastohydrodynamic Contacts. J. Tribol..

[49] Gohar R., Rahnejat H. (2008). Fundamentals of Tribology.

[50] Greenwood J.A., Tripp J.H. (1970). The Contact of Two Nominally Flat Rough Surfaces. Proc. Inst. Mech. Eng..

[51] Teodorescu M., Kushwaha M., Rahnejat H., Rothberg S.J. (2007). Multi-physics analysis of valve train systems: From system level to microscale interactions. Proc. Inst. Mech. Eng. Part K J. Multi-Body Dyn..

[52] Zavos A., Nikolakopoulos P.G. (2018). Tribology of new thin compression ring of fired engine under controlled conditions-A combined experimental and numerical study. Tribol. Int..

[53] Baker C., Rahmani R., Theodossiades S., Rahnejat H., Fitzsimons B. (2015). On the Effect of Transient In-Plane Dynamics of the Compression Ring Upon Its Tribological Performance. J. Eng. Gas Turbines Power.

[54] Morris N., Mohammadpour M., Rahmani R., Rahnejat H. (2017). Optimisation of the piston compression ring for improved energy efficiency of high performance race engines. Proc. Inst. Mech. Eng. Part D J. Automob. Eng..

[55] Taylor R., Morgan N., Mainwaring R., Davenport T. (2020). How much mixed/boundary friction is there in an engine—And where is it?. Proc. Inst. Mech. Eng. Part J J. Eng. Tribol..

[56] Koch F., Geiger U., Hermsen F.-G. (1996). PIFFO—Piston Friction Force Measurements during Engine Operation.

[57] Stępień Z. (2021). A Comprehensive Overview of Hydrogen-Fueled Internal Combustion Engines: Achievements and Future Challenges. Energies.

[58] Yip H.L., Srna A., Yuen A.C.Y., Kook S., Taylor R.A., Yeoh G.H., Medwell P.R., Chan Q.N. (2019). A Review of Hydrogen Direct Injection for Internal Combustion Engines: Towards Carbon-Free Combustion. Appl. Sci..

[59] Verhelst S. (2014). Recent progress in the use of hydrogen as a fuel for internal combustion engines. Int. J. Hydrogen Energy.

[60] Ansys Granta, Ansys Granta Selector—Materials Selection Software, (2023 R2). https://www.ansys.com/products/materials/granta-selector.

[61] Dobrucali E. (2016). The effects of the engine design and running parameters on the performance of a Otto–Miller Cycle engine. Energy.

